# Immunogenicity and cross-reactivity against *Mycobacterium tuberculosis* of proteoliposomes derived from *Mycobacterium bovis* BCG

**DOI:** 10.1186/1471-2172-14-S1-S7

**Published:** 2013-02-25

**Authors:** Fátima Reyes, Yanely Tirado, Alina Puig, Reinier Borrero, Giselle Reyes, Sonsire Fernández, José Luis Pérez, Ramlah Kadir, Caridad Zayas, Mohd Nor Norazmi, María E Sarmiento, Armando Acosta

**Affiliations:** 1 Finlay Institute. Ave. 27 No. 19805, Havana, Cuba. AP. 16017, CP11600; 2 School of Health Sciences, Universiti Sains Malaysia, 16150 Kubang Kerian, Malaysia; 3 Institute for Research in Molecular Medicine, Universiti Sains Malaysia, 16150 Kubang Kerian, Malaysia

## Abstract

The only currently available vaccine against tuberculosis (TB) is *Mycobacterium bovis* Bacille Calmette-Guerin (BCG), which has inconsistent efficacy to protect against the disease in adults. *M. tuberculosis* (MTB) cell wall components have been implicated in the pathogenicity of TB and therefore have been a prime target for the identification and characterization of cell wall proteins with potential application in vaccine development. In this regard, proteoliposomes (PLs) derived from mycobacteria containing lipids and cell wall proteins could be potential vaccine candidates against TB. In the present study PLs derived from BCG were prepared. These homogeneous population of spherical microparticles was then immunized into Balb/c mice. Sera of immunized animals showed high IgG response and strong cross-reactivity against different MTB antigens.These results showed that BCG PLs could be potential vaccine candidates against TB.

## Introduction

TB remains a major infectious disease which causes high morbidity and mortality worldwide [1]. BCG, an attenuated strain of *Mycobacterium bovis* (Mb), is the current vaccine approved for human use against TB. It is most effective in protecting children from the disease; while its efficacy in adults is poor especially against pulmonary TB, proving the prevailing necessity to obtain a more effective vaccine [2].

The availability of the complete genome sequence of MTB and Mb, as well as access to different programs for epitope prediction and homology search between sequences, allows the identification of conserved proteins among both species with the potential of identifying components for new vaccines [3]. Additionally, many studies support the role of mycobacterial cell wall components in the development of TB pathogenesis [4].

Since mycobacterial cell wall components have been suggested to be potential targets for the development of new TB vaccine formulations, we attempted to use PLs from BCG to determine the immunogenicity and cross-reactivity of these microparticles against MTB. The presence of proteins in the PLs is expected to enhance the immune response against MTB.

## Materials and methods

### *In silico* assays

Proteins with possible localization in the cell wall of MTB were retrieved from the scientific literature. From a total of 306 MTB cell wall proteins a set of five immunodominant and immunogenic proteins (Acr, Ag85B, Mce1A, HbhA and L7/L12) were selected for in silico alignment with the corresponding proteins in Mb using ClustalW (http://www.ebi.ac.uk/Tools/msa/clustalw2/).

Prediction of B cell epitopes of these proteins was performed by using ABCpred server (http://www.imtech.res.in/raghava/abcpred/). Selected B cell epitopes were synthesized in the Centre for Genetic Engineering and Biotechnology (CIGB), Cuba, and were used in the immunogenicity assay.

### Preparation and partial characterization of PLs derived from Mb BCG (BCG PLs)

BCG PLs were prepared according to the methodology described by Rodriguez et al [5]. Molecular size of PLs was determined by size exclusion chromatography using an XK 16/100 column packed with Sephacryl S-1000 (Pharmacia). The morphology of BCG PLs was determined by Transmission Electron Microscopy (TEM) with negative staining.

### Immunogenicity and cross-reactivity assays

Eighteen male Balb/c mice (5-6 weeks), supplied by the School of Health Sciences (Universiti Sains Malaysia, Malaysia) were used in the experiments. All procedures were carried out according to the international regulations of laboratory animal experimentation [6] and approved by the USM Animal Ethics Committee.

Three groups of animals (n=6 per group) were inoculated with either 100 µl PBS (negative control), BCG (10^6^ CFU) or BCG PLs (50 mg + Freund's Incomplete Adjuvant) by the intraperitoneal route. Mice were immunized at days zero and 21 and bled for serum collection at days zero, 14, 28 and 35. Sera samples from each group were stored at -20 °C for subsequent analysis.

Humoral immune response and cross-reactivity against MTB were evaluated by indirect ELISA [5]. Coating antigens comprised BCG whole cells (10^6^ CFU/mL), five B cell epitopes from MTB proteins or MTB antigens such as cell wall fraction (CWF), soluble cell wall proteins (SCWP), lipoarabinomanan (LAM) and purified protein derivative (PPD). These antigens were kindly provided by BEI Resources, ATCC, USA.

### Statistical analysis

Data from the *in vitro* assays were analyzed for statistical significance using a simple ANOVA test. Duncan's multiple range test and Tukey's Post Hoc Test were used for the determination of pairs with significant differences.

## Results and discussion

### *In silico* assays

MTB cell wall proteins have been suggested to contain virulence factors [4]. Since the BCG genome is more than 90% homologous to that of MTB [7], it is reasonable to assume that BCG PLs can be potential vaccine candidates against TB.

The five MTB cell wall proteins selected in our study are 100% identical to their corresponding proteins in BCG (data not shown). This could explain the partial protection afforded by BCG vaccination in humans [8].

### Preparation and partial characterization of BCG PLs

The molecular size of BCG PLs was estimated by the coefficient of Ve/Vt relation obtained from size exclusion chromatography. This coefficient was 0.45, indicating that the molecular size of PLs was above 100 nm, using as reference the characterization studies of VAMENGOC-BC vaccine and *V. cholerae* PLs [9,10]. The morphology of BCG PLs as assessed by TEM showed a homogeneous population of spherical structures (results not shown). The molecular size of BCG PLs was also confirmed by TEM.

### Immunogenicity study

**Figure 1:**
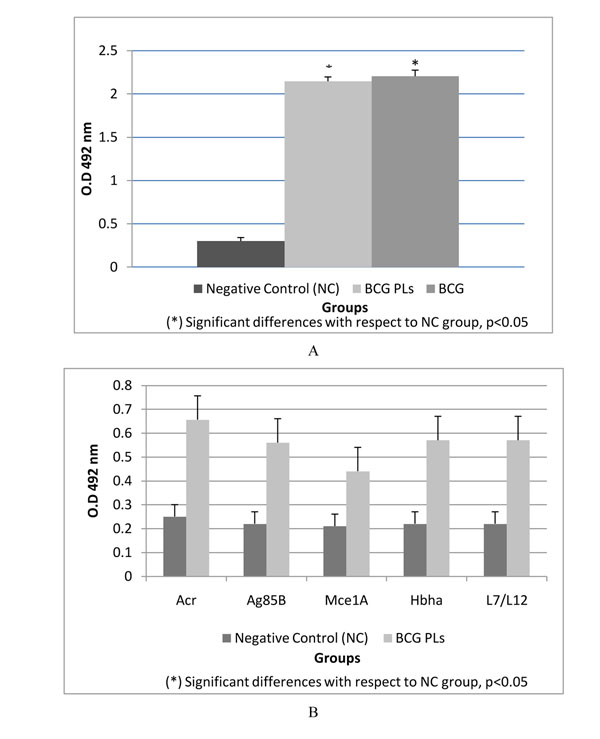
**Antigen recognition and cross reactivity induced by BCG PLs immunization. ELISA. A** Reactivity of total IgG from mice immunized with BCG PLs or BCG to BCG antigens (whole cells). **B** Reactivity of total IgG from mice immunized with BCG PLs against B epitopes from MTB. The statistical analysis was performed by one-way ANOVA followed by Tukey post-hoc test. (*) significant differences with respect to NC group, p < 0.05

There were no statistically significant differences among groups of mice immunized with BCG PLs or BCG. However both groups induced significantly (p<0.05) higher IgG levels against BCG whole cells than those inoculated with PBS (Fig. 1A).

In addition, animals immunized with BCG PLs produced significantly (p<0.05) higher IgG levels against B cell epitopes compared to those from animals inoculated with PBS (Fig. 1B).

Proteoliposomes from bacteria contain LPS, proteins and other molecules that are known as pathogen-associated molecular patterns (PAMPs), which possess immune enhancing, and modulator effects [9]. The interaction between cell wall proteins, lipids and detergent molecules leads to the extraction and formation of spherical nanoparticles or proteoliposomes [9]. Our results suggest the presence of the five B cell epitopes from MTB in the BCG PLs (Fig.1B). The epitopes identified *in silico* belong to proteins, which are involved in the pathogenicity of MTB. Therefore, these antigens could be used to stimulate a protective response against TB.

For example, the Acr protein is a major MTB antigen recognized by the sera of a high proportion of TB patients [11]. Recognition of B cell epitope of Ag85B may have significance for protection as this protein is related to the interaction with fibronectin, a key element of the invasion of tissues by MTB [12]. Moreover, Mce1A protein plays an important role in macrophage invasion [13], so Mce1A-antibodies could affect the entry of mycobacteria into the cell. HbhA protein is involved in extrapulmonary dissemination of MTB [14]. Immune response against these B cell epitopes could prevent the development of extrapulmonaryTB.

### Cross-reactivity study

**Figure 2:**
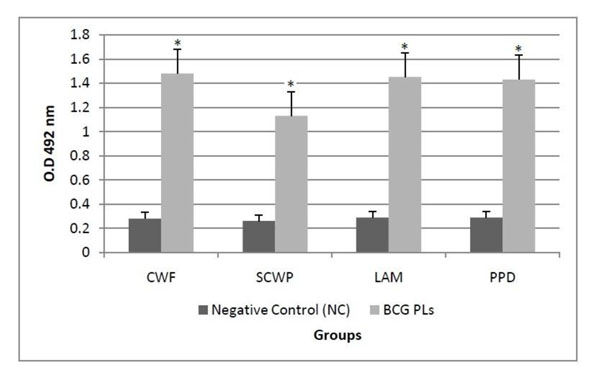
**Cross-reactivity against MTB antigens. ELISA.** Reactivity of total IgG from mice immunised with BCG PLs against MTB antigens. The statistical analysis was performed by a one-way ANOVA followed by Tukey post-hoc test. CWF: cell wall fraction, SCWP: soluble cell wall proteins, LAM: lipoarabinomannan, PPD: purified protein derivative. (*) significant differences with respect to NC group, p < 0.05

Sera from mice immunized with BCG PLs showed specific IgG response against all MTB antigens used (Fig. 2). This response was significantly (p<0.01) higher compared to animals inoculated with PBS. The induction of specific humoral immune response recognizing MTB antigens after the immunization with BCG PLs is interesting considering the potential role of antibodies in the protection against TB [1]. In addition, this result demonstrates the presence of other relevant MTB antigens in BCG PLs, which could be involved in inducing cross-protective immunity against TB.

## Conclusions

In this paper we selected *in silico* a group of MTB cell wall proteins which posses 100% identity with its counterparts in Mb BCG. These proteins were positively recognised by sera of mice immunized with BCG PLs obtained by detergent extraction method. This result suggests that BCG PLs contain the highly conserved proteins, which also induce a strong humoral immune response and could generate a cross-protective response to MTB infection.

## Competing interests

The authors declare that they have no competing financial interests.

## Authors' contributions

All authors have read and approved the final manuscript. FR and YT participated in the bioinformatics studies, immunogenicity and cross reactivity studies, data analyses and in writing of the manuscript. AP participated in the production of PLs and in performing the immunogenicity and cross reactivity studies. RB, RK and CZ performed the immunogenicity and cross reactivity studies. GR, SF and JLP worked on the production and characterization of PLs. MNN, MES and AA conceived the study, participated in bioinformatics studies, data analyses and in writing and finalizing of the manuscript.

## Declarations

This article has been published as part of *BMC Immunology* Volume 14 Supplement 1, 2013: Proceedings from Delivery Systems and Current strategies to drug design. The full contents of the supplement are available online at http://www.biomedcentral.com/bmcimmunol/supplements/14/S1.

## References

[B1] Acosta A Norazmi MN Sarmiento ME Antibody mediated immunity - a missed opportunity in the fight against tuberculosis? Malays J Med Sci 17 2 66 67 22135541 PMC3216152

[B2] Martín C Bigi F Gicquel B New Vaccines against Tuberculosis Tuberculosis 2007: From basic science to patient care 2007 First 341 360

[B3] Dietrich J Weldingh K Andersen P Prospects for a novel vaccine against tuberculosis Vet Microbiol 2006 112 2-4 163 169 10.1016/j.vetmic.2005.11.030 16325357

[B4] Smith I Mycobacterium tuberculosis pathogenesis and molecular determinants of virulence Clin Microbiol Rev 2003 16 3 463 496 10.1128/CMR.16.3.463-496.2003 12857778 PMC164219

[B5] Rodriguez L Tirado Y Reyes F Puig A Kadir R Borrero R Fernandez S Reyes G Alvarez N Garcia MA Proteoliposomes from Mycobacterium smegmatis induce immune cross-reactivity against Mycobacterium tuberculosis antigens in mice Vaccine 2011 29 37 6236 6241 10.1016/j.vaccine.2011.06.077 21736914

[B6] Canadian Council On Animal Care Guide to the care and use of experimental animals 1984 Ottawa, Ont

[B7] Garnier T Eiglmeier K Camus JC Medina N Mansoor H Pryor M Duthoy S Grondin S Lacroix C Monsempe C The complete genome sequence of Mycobacterium bovis Proc Natl Acad Sci U S A 2003 100 13 7877 7882 10.1073/pnas.1130426100 12788972 PMC164681

[B8] Andersen P Doherty TM The success and failure of BCG - implications for a novel tuberculosis vaccine Nat Rev Microbiol 2005 3 8 656 662 10.1038/nrmicro1211 16012514

[B9] Perez JL Acevedo R Callico A Fernandez Y Cedre B Ano G Gonzalez L Falero G Talavera A Perez O A proteoliposome based formulation administered by the nasal route produces vibriocidal antibodies against El Tor Ogawa Vibrio cholerae O1 in BALB/c mice Vaccine 2009 27 2 205 212 10.1016/j.vaccine.2008.10.052 18996426

[B10] Campa C Sierra VG Gutierrez MM Biset G García LG Puentes G Method of producing Neisseria meningitidis B vaccine, and vaccine produced by method 1997 US 597,572

[B11] Yuan Y Crane DD Simpson RM Zhu YQ Hickey MJ Sherman DR Barry CE 3rd The 16-kDa alpha-crystallin (Acr) protein of Mycobacterium tuberculosis is required for growth in macrophages Proc Natl Acad Sci U S A 1998 95 16 9578 9583 10.1073/pnas.95.16.9578 9689123 PMC21381

[B12] Kremer L Maughan WN Wilson RA Dover LG Besra GS The M. tuberculosis antigen 85 complex and mycolyltransferase activity Lett Appl Microbiol 2002 34 4 233 237 10.1046/j.1472-765x.2002.01091.x 11940150

[B13] Haile Y Caugant DA Bjune G Wiker HG Mycobacterium tuberculosis mammalian cell entry operon (mce) homologs in Mycobacterium other than tuberculosis (MOTT) FEMS Immunol Med Microbiol 2002 33 2 125 132 10.1111/j.1574-695X.2002.tb00581.x 12052567

[B14] Pethe K Alonso S Biet F Delogu G Brennan MJ Locht C Menozzi FD The heparin-binding haemagglutinin of M. tuberculosis is required for extrapulmonary dissemination Nature 2001 412 6843 190 194 10.1038/35084083 11449276

